# USP17L13 Enhances Influenza a Virus Replication by Mediating the Degradation of RIG-I and MDA5

**DOI:** 10.3390/v18050575

**Published:** 2026-05-20

**Authors:** Yaping Zhang, Chen Qin, Yichao Zhuang, Lei Chen, Xianying Zeng, Li Jiang, Chengjun Li, Hualan Chen, Huihui Kong

**Affiliations:** State Key Laboratory of Animal Disease Control and Prevention, Harbin Veterinary Research Institute, Chinese Academy of Agricultural Sciences, Harbin 150069, China; zyp412321@163.com (Y.Z.); qinchen0910@126.com (C.Q.); zhuangachao@yeah.net (Y.Z.); clyywd@163.com (L.C.); zengxianying@caas.cn (X.Z.); jiangli@caas.cn (L.J.); lichengjun@caas.cn (C.L.)

**Keywords:** influenza, USP17L13, IFN, RIG-I, MDA5

## Abstract

The innate immune system, particularly the retinoic acid-inducible gene I (RIG-I)-like receptor (RLR) signaling pathway, is a major early defense barrier against influenza A virus infection. However, excessive immune responses can trigger lethal cytokine storms and severe immune-mediated pathology. In this study, we performed a genome-wide CRISPR/dCas9 gene activation screen in human lung epithelial (A549) cells by using an A/Puerto Rico/8/1934 (H1N1) reporter virus, and identified the ubiquitin-specific protease USP17L13 as a novel negative regulator of innate immunity that promotes influenza virus replication. Overexpression of USP17L13 significantly enhanced the replication of multiple subtypes of influenza viruses in A549 cells, including a human pandemic H1N1 virus, seasonal H3N2 viruses, as well as a globally circulating clade, 2.3.4.4b, of the highly pathogenic avian H5N1 virus. Transcriptomic analysis demonstrated that USP17L13 suppresses host antiviral defenses by downregulating nuclear factor kappa B (NF-κB) signaling and arachidonic acid metabolism, while upregulating pathways associated with ribosomal translation and oxidative phosphorylation to facilitate viral production. Mechanistically, USP17L13 attenuates the host interferon (IFN) response by promoting the degradation of the key viral RNA sensors, RIG-I, and melanoma differentiation-associated protein 5 (MDA5). Further analysis revealed that USP17L13 is inducible by type I and type II interferons as well as inflammatory cytokines, suggesting that it may act as a negative-feedback regulator to limit excessive inflammation. Collectively, our findings identify USP17L13 as a previously unrecognized proviral host factor and provide new insight into how host deubiquitinases shape influenza virus-host interactions, with potential implications for host-directed approaches to controlling excessive inflammation during viral infection and improving influenza vaccine production.

## 1. Introduction

Influenza A virus, a member of the family *Orthomyxoviridae*, is a pathogen with a segmented, negative-sense, single-stranded RNA genome. Historically, influenza A virus subtypes, including H1, H2, and H3, were responsible for four influenza pandemics in 1918, 1957, 1968, and 2009 [1,2], resulting in about 50 million deaths worldwide. Currently, H1 and H3 viruses continue to circulate in humans as seasonal influenza viruses, leading to approximately 3–5 million severe infections and up to 650,000 annual fatalities globally [3]. In addition, avian influenza viruses have sporadically crossed the species barrier to infect humans, with H5N1 and H7N9 representing two of the most important zoonotic subtypes and posing serious public health concerns, causing 3040 human infections and over 1137 deaths [4,5,6,7,8,9]. The outcome of influenza virus infection reflects a dynamic interplay between viral replication and host responses. Upon entering host cells, the virus must evade and counteract innate immune defenses to establish productive infection. Meanwhile, influenza virus infection can trigger excessive inflammation and immune-mediated pathology, which are major contributors to disease severity and mortality. In particular, dysregulated cytokine responses and excessive inflammatory activation are closely associated with severe influenza-associated immunopathology [10,11,12].

After binding to attachment receptors, such as sialic acid receptors, and entering cells [13,14,15], influenza A viral RNA is recognized by pattern recognition receptors (PRRs), which trigger innate immune responses. Among these PRRs, retinoic acid-inducible gene I (RIG-I) acts as the principal RIG-I-like receptor (RLR) [16], whereas melanoma differentiation-associated protein 5 (MDA5) plays a complementary role [17]. The activity of this antiviral signaling pathway is tightly controlled by post-translational modifications and protein turnover, through both the ubiquitin–proteasome system and the autophagy-lysosomal pathway [18]. Accumulating evidence indicates that deubiquitinating enzymes (DUBs) play dual roles during influenza virus infection [19]. Ubiquitin-specific peptidase 1 (USP1) enhances RIG-I stability through deubiquitination, leading to increased production of inflammatory cytokines and consequent inhibition of viral replication [20]. Similarly, USP4 promotes the stability of RIG-I and mitochondrial antiviral-signaling protein (MAVS) and stimulates interferon-β (IFN-β) production, thereby suppressing viral replication [21,22]. USP25 confers antiviral protection by promoting the synthesis of proinflammatory cytokines and type I IFN. This regulatory effect is achieved by deubiquitinating tumor necrosis factor receptor-associated factor 3 (TRAF3) by removing K48-linked chains and by stabilizing TRAF6 by removing K48-linked chains [23]. In contrast, USP11 deubiquitinates nucleoprotein (NP) and inhibits the activity of the influenza viral ribonucleoprotein complex [24]. By comparison, USP39 stabilizes the viral PB2 protein and thereby promotes viral replication [25]. Interestingly, USP18 deubiquitinates cyclic GMP-AMP synthase (cGAS) and promotes cytokine production, but it also enhances apoptosis, which may ultimately facilitate influenza virus replication [26]. Collectively, these findings indicate that DUBs regulate distinct steps of the virus–host interaction and may either suppress or promote influenza virus replication depending on the specific target.

The USP17 family, also referred to as the DUB3 or USP17-like family, consists of 26 highly homologous deubiquitinating enzymes. However, the functions of most members of this family have not yet been systematically characterized. Among these proteins, USP17L2 is one of the best-studied members and has been implicated in the regulation of multiple cancer-related and innate immune response pathways. For example, USP17L2 has been reported to stabilize Yes-associated protein 1 (YAP1), thereby promoting the development and progression of hepatocellular carcinoma, colorectal cancer, and ovarian cancer [27]. Acevedo et al. further showed that USP17L2 removes ubiquitin from β-catenin, activates Wingless/Integrated (Wnt)/β-catenin signaling, and consequently drives colorectal cancer progression [28]. In another study, Niki et al. demonstrated that USP17L2 cooperates with USP4 to deubiquitinate platelet-derived growth factor receptor β (PDGFRβ), regulate its trafficking, and enhance the signal transducer and activator of transcription 3 (STAT3)-dependent transcription, ultimately promoting cell proliferation [29]. USP17L2 has also been linked to innate immune regulation. Chen et al. found that knockdown of endogenous USP17L2 markedly increases the ubiquitination of the viral RNA sensors RIG-I and MDA5 [30], thereby promoting the replication of vesicular stomatitis virus (VSV). Collectively, these studies highlight the functional importance of USP17 family members, while the biological roles of most other homologous proteins remain largely unexplored.

In this study, to elucidate the roles of the broader USP17 family during influenza virus infection, we screened several USP17 homologs that had been identified as potential proviral host factors. Through systematic mechanistic investigations, we found that USP17L13 is the most potent member in terms of promoting influenza virus replication and acts through mechanisms distinct from those of the best-studied family member, USP17L2. Our findings reveal a previously unrecognized proviral role for USP17L13, provide new insight into its immunomodulatory function during viral infection, and offer a basis for exploring host-directed strategies to modulate excessive virus-induced inflammation and improving influenza vaccine production.

## 2. Materials and Methods

### 2.1. Biosafety Facilities

All procedures involving live virus handling were performed in our biosafety level 2 (BSL-2) laboratory or at the BSL-3 facility of the Harbin Veterinary Research Institute, Chinese Academy of Agricultural Sciences.

### 2.2. Viruses and Cells

The low pathogenic influenza viruses used in this study, including A/Puerto Rico/8/1934 (H1N1, PR8), A/Sichuan/1/2009 (H1N1), and A/Kansas/14/2017 (H3N2), and the highly pathogenic avian influenza virus A/duck/Guizhou/S1194/2023 (H5N1), were maintained in our laboratory. The PR8 reporter virus, which carries a Venus fluorescent gene inserted into the NS segment and was generated as previously described [31], is preserved in our laboratory. The H1 and H3 subtype viruses were amplified in MDCK (Madin–Darby canine kidney; ATCC CCL-34) cells, whereas the H5 virus was amplified in embryonated chicken eggs (10 days old). MDCK, HEK293T (human embryonic kidney 293T; ATCC CRL-3216), and A549 (human lung epithelial; ATCC CCL-185) cells were purchased from ATCC (Manassas, VA, USA) and maintained in our laboratory. MDCK cells were cultured in DMEM (Dulbecco’s modified Eagle’s medium) supplemented with 6% NCS (newborn calf serum), whereas HEK293T and A549 cells were cultured in DMEM supplemented with 8% FBS (fetal bovine serum). All cell lines were maintained at 37 °C in an atmosphere containing 5% CO_2_.

### 2.3. Generation and Screening of the CRISPR Activation Library

A gene-overexpression library in A549 cells was established using the human CRISPR activation library (Addgene, Watertown, MA, USA) as previously described by Joung et al. [32] To screen for host factors that facilitate influenza virus replication, 1 × 10^7^ library cells were infected with PR8 reporter influenza virus at an MOI of 10. At 12 h post infection, cells were sorted by fluorescence-activated cell sorting (Beckman, Brea, CA, USA), and the top 1% of cells with the highest fluorescence intensity were collected for next-generation sequencing.

### 2.4. Phylogenetic Analysis of USP17 Family Homologs

A total of 26 human USP17 homologs were downloaded from the public available database (https://www.ncbi.nlm.nih.gov/), URL (accessed on 8 May 2024). The amino acid sequences were aligned using the Clustal W algorithm, and a maximum likelihood phylogenetic tree was generated using MEGA (11.0.13).

### 2.5. Transcriptomic Analysis

To evaluate the host cellular transcriptomic landscape, RNA sequencing was performed on A549 cells with or without stable USP17L13 overexpression. The cells were infected with the PR8 virus at an MOI of 1 and harvested at 12 h post infection for subsequent RNA sequencing. To systematically assess host gene expression changes (Table S3), Gene Ontology (GO) enrichment analysis, including the molecular function (MF), cellular component (CC), and biological process (BP) categories, was conducted alongside Kyoto Encyclopedia of Genes and Genomes (KEGG) pathway analysis and gene set enrichment analysis (GSEA).

### 2.6. Generation of Cells Overexpressing USP17 Homologs

To overexpress USP17 homologs, the coding sequences of USP17L2, USP17L7, USP17L10, and USP17L13, each fused with a C-terminal Flag tag, were synthesized and cloned into a lentiviral vector using Gibson assembly (Vazyme, Nanjing, China). For lentivirus packaging, HEK293T cells were co-transfected with the lentiviral transfer plasmid together with psPAX2 and pVSV-G at a ratio of 4:2:1. The culture medium containing lentiviral particles was collected 48 h later and used for transduction of A549 cells. To ensure that each A549 cell was infected by only one lentivirus, A549 cells were transduced at a low MOI (0.1–0.3). After 24 h of incubation, the transduced cells were selected with puromycin (2 μg/mL). The surviving cells were used for further experimental analyses.

### 2.7. Growth Kinetics of Influenza Viruses in A549 Cells Overexpressing USP17 Homologs

To determine the effects of USP17 homologs on influenza virus replication, A549 cells overexpressing USP17L2, USP17L7, USP17L10, USP17L13, or the empty vector were infected with PR8 virus at an MOI of 0.01 and incubated at 37 °C. To evaluate the effect of USP17L13 on the replication of different influenza virus subtypes, A549 cells with or without USP17L13 overexpression were infected with H1, H3, and H5 viruses at an MOI of 0.01. For the human H1 and H3 influenza viruses, infected cells were maintained at 35 °C to mimic the temperature of the human upper respiratory tract. For the H5 avian influenza virus, infected cells were maintained at 37 °C, which is closer to the temperature of the lower respiratory tract. Supernatants were collected from infected cell cultures at the specified time points for determination of viral titers. Virus titers were determined in MDCK cells by plaque assay. Briefly, 100% confluent MDCK monolayers were washed twice with PBS and inoculated with 10-fold serially diluted samples at room temperature. After 1 h of infection, the supernatant was removed, and the cells were overlaid with minimum essential medium (MEM) containing 0.8% SeaPlaque agarose (Lonza, Morristown, NJ, USA), 0.3% bovine serum albumin (BSA), and 1 μg/mL TPCK-treated trypsin (L-1-tosylamido-2-phenylethyl chloromethyl ketone-treated trypsin (Sigma, Burlington, MA, USA). The cells were then incubated for 48 h before plaque counting.

### 2.8. VSV-GFP Reporter Assay

To evaluate IFN production in A549 cells, cells overexpressing USP17L13, USP17L2, or the vector control were infected with VSV-GFP at doses of 4, 2, 1, or 0.5 TCID_50_. At 36 h post infection, infection was assessed by fluorescence microscopy.

### 2.9. RT-qPCR

To quantify IFN-β mRNA induction by poly(I: C) and poly(dA: dT), which mimic dsRNA and dsDNA, respectively, A549 cells overexpressing USP17L2, USP17L13, or an empty vector were transfected with 1.5 μg of poly(I: C) or poly(dA: dT). At 48 h post transfection, total RNA was extracted from cells using TRIzol reagent, and reverse transcription was performed using the PrimeScript RT reagent Kit with gDNA Eraser (Takara, Shiga Prefecture, Otsu, Japan) according to the manufacturer’s instructions. Briefly, residual genomic DNA was removed at 42 °C for 2 min, followed by reverse transcription at 37 °C for 15 min and 85 °C for 5 s. Quantitative PCR was performed using TB Green Premix Ex Taq II (Takara, Shiga Prefecture, Otsu, Japan). The cycling conditions were as follows: 95 °C for 30 s, followed by 40 cycles of 95 °C for 5 s and 60 °C for 34 s. Melt curve analysis was performed to confirm amplification specificity. IFN-β mRNA expression was assessed by RT-qPCR using designed primers (Homo-IFN-β forward, CATTACCTGAAGGCCAAGGA; Homo-IFN-β reverse, CAATTGTCCAGTCCCAGAGG). Homo-β-actin was used as an internal control using primers (Homo-β-actin forward: AGAGCTACGAGCTGCCTGAC; and Homo-β-actin reverse, AGCACTGTGTTGGCGTACAG).

### 2.10. Protein Degradation Assay

Protein degradation was analyzed by Western blotting. To evaluate the specific degradative effects on key factors in the IFN signaling pathway, HEK293T cells were co-transfected with expression plasmids encoding the target proteins [RIG-I-HA, MDA5-HA, MAVS-HA, inhibitor of nuclear factor kappa-B kinase subunit epsilon ε (IKKε)-Flag, and stimulator of interferon genes (STING)-Myc], together with either USP17L13-Flag, USP17L2-Flag (used as a comparative control), or an empty vector. At 48 h post transfection, cells were harvested, and total cellular proteins were extracted for analysis. The expression of the target proteins was then determined by Western blotting using specific primary antibodies, including anti-HA, anti-Flag, and anti-Myc. For the analysis of RIG-I-HA, MDA5-HA, MAVS-HA, and IKKε-Flag, glyceraldehyde-3-phosphate dehydrogenase (GAPDH) was used as a control, whereas Tubulin was used as an internal control for STING-Myc analysis. IRDye^®^ 800CW Goat anti-Mouse IgG secondary antibody was used for detection (Thermo Fisher Scientific, Waltham, MA, USA). Relative protein levels were analyzed by densitometric quantification and normalized to the corresponding GAPDH or Tubulin control.

### 2.11. Immune Stimulation Assay

To examine the induction of USP17L13 expression by interferons and inflammatory cytokines, wild-type A549 cells were treated with the indicated purified proteins for 36 h, including IFN-α, IFN-β, IFN-ω, and IFN-γ at 5000 U/mL, TNF-α at 20 ng/mL, and IL-4 at 50 ng/mL. Then, total RNA was extracted, and USP17L13 mRNA levels were analyzed by qPCR using designed primers (Forward: CTTGTGATCCTTGTTCCAGT, Reverse: ACATGAGAGTGGTGACTTCT).

#### Statistical Analysis

Statistical significance was determined using independent *t*-tests or two-way ANOVA, as appropriate for the experimental design, in GraphPad Prism (9.3). Significance was inferred when *p* values did not exceed 0.05.

## 3. Results

### 3.1. USP17 Family Members Promote Influenza Virus Replication

**Figure 1:**
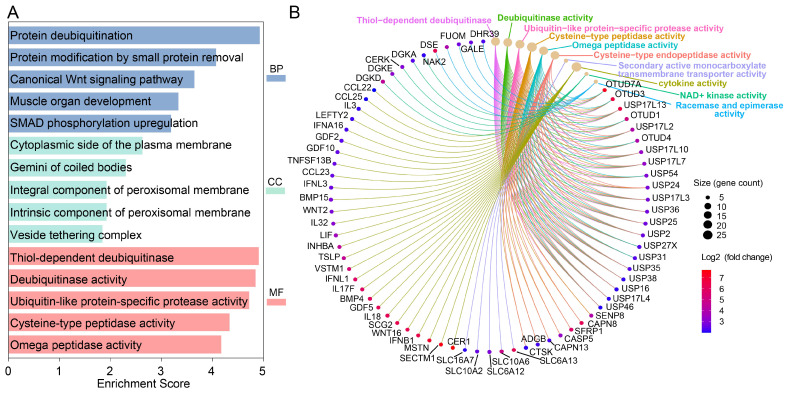
Gene ontology (GO) enrichment analysis of differentially expressed genes. (**A**) Distribution of significantly enriched GO terms. The horizontal bar plot displays the top 10 enriched functional categories categorized into three domains: Molecular Function (MF, pink), Cellular Component (CC, green), and Biological Process (BP, blue). The x-axis represents the enrichment score, indicating the degree of over-representation for each term. (**B**) Gene-concept network (cnetplot) of key functional pathways. This network illustrates the linkages between specific differentially expressed genes (DEGs) and their associated GO terms.

To identify host factors that may promote influenza virus replication, we generated a gene-overexpression cell library in human lung epithelial A549 cells using the human CRISPR activation library [32]. We then screened this library with the A/Puerto Rico/8/1934 (H1N1, PR8) reporter virus carrying a Venus fluorescent gene inserted into the NS segment, generated as previously described [31]. PR8 is a mouse-adapted influenza A virus strain widely used as a model virus for studying influenza virus replication, pathogenesis, and host-virus interactions. After infection at a multiplicity of infection (MOI) of 10, infected cells were sorted by fluorescence-activated cell sorting, and the top 1% of cells with the strongest fluorescent signals were collected for next-generation sequencing. After sequencing, genes enriched by more than 2 log_2_-fold were subjected to gene ontology (GO) enrichment analysis across three categories: biological process (BP), molecular function (MF), and cellular component (CC) (Figure 1A and Table S1). In the BP category, these genes were markedly enriched in pathways linked to protein deubiquitination, protein modification by small protein removal, and the canonical Wnt signaling. In the MF category, genes associated with thiol-dependent deubiquitinase, deubiquitinase activity and ubiquitin-like protein-specific protease activity were prominently enriched. Consistently, the cnetplot analysis, which displays the connections between functions and genes, highlighted a cluster of USP17 family members, including USP17L2, USP17L3, USP17L4, USP17L7, USP17L10, and USP17L13, as central nodes within the deubiquitination-related network in the MF analysis (Figure 1B). For comparison, we also analyzed the data from King et al. and identified similar host genes involved in deubiquitinase activity that facilitate influenza virus replication through a pathogen-driven CRISPR activation screen [33] (Figure S1A), including USP17L2, USP17L3, USP17L7, USP17L8, USP17L10 and USP17L13 (Figure S1B and Table S2). Together, these observations consistently suggest that USP17 family members may participate in key stages of the influenza virus life cycle.

### 3.2. USP17L13 Acts as a Potent Proviral Factor Across Multiple Influenza Viruses

**Figure 2:**
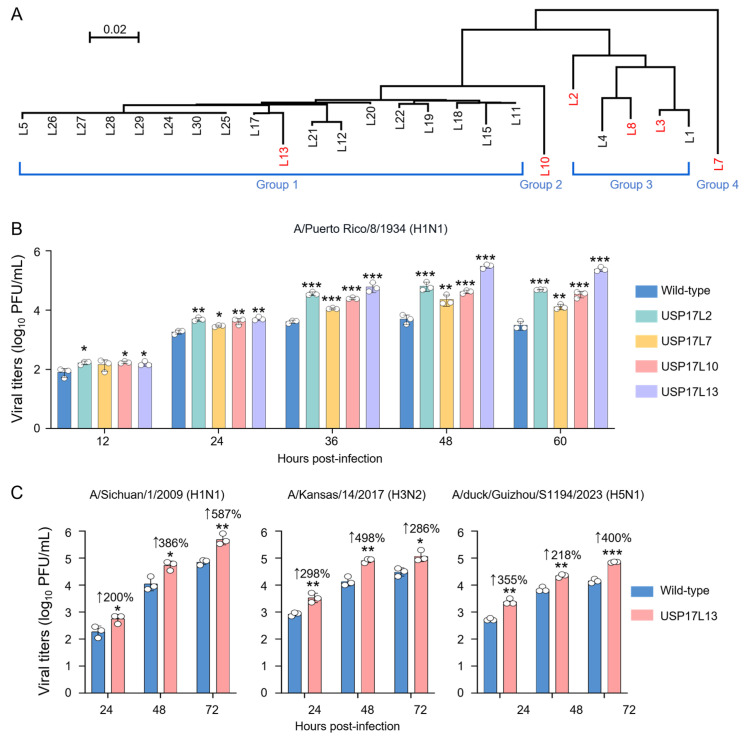
USP17L13 promotes the replication of diverse influenza virus. (**A**) Phylogenetic analysis of human USP17 homologs. A phylogenetic tree was constructed using the amino acid sequences of 26 human USP17 homologs. The homologs are categorized into four distinct evolutionary clusters (Groups 1–4). (**B**) Growth kinetics of A/Puerto Rico/8/1934 (H1N1) virus in A549 cells expressing different USP17 homologs. A549 cells stably expressing C-terminal Flag-tagged USP17L2, USP17L7, USP17L10, or USP17L13 were generated via lentiviral transduction and puromycin selection. Cells were infected with the indicated virus at a multiplicity of infection (MOI) of 0.01. Viral titers in the culture supernatants were determined by plaque assay. (**C**) USP17L13 enhances the replication of multiple subtypes of influenza virus. A549 cells stably expressing USP17L13 or a wild-type control were infected with the indicated virus at an MOI of 0.01. Supernatants were for viral titration. Data are presented as mean ± SD (*n* = 3). Statistical significance was determined by two-way ANOVA (* *p* < 0.05; ** *p* < 0.01; *** *p* < 0.001).

To determine the functional role of the USP17 family during influenza virus replication, we first performed a comprehensive phylogenetic analysis of 26 USP17 homologs downloaded from public databases. Based on their distribution across four major evolutionary groups, four representative candidates, USP17L2, USP17L7, USP17L10, and USP17L13, were chosen for subsequent functional analyses (Figure 2A).

These genes, harboring a C-terminal Flag tag, were stably transduced into A549 cells via a lentiviral delivery system. We first evaluated the impact of these homologs on the replication kinetics of PR8. The A549 cell populations that survived after puromycin selection were infected with PR8 virus at a multiplicity of infection (MOI) of 0.01, and the supernatants from different time points post infection were collected for titration. As shown in Figure 2B, all four tested USP17 homologs significantly enhanced PR8 replication to varying degrees compared to the wild-type control. Notably, USP17L13 exhibited the most robust proviral activity. To determine whether this promoting effect extends to a broader range of influenza viruses, we further assessed the impact of USP17L13 on the replication of diverse human and avian-origin strains, including a 2009 pandemic H1N1 (A/Sichuan/1/2009), a seasonal H3N2 (A/Kansas/14/2017), and a highly pathogenic avian influenza H5N1 strain (A/duck/Guizhou/S1194/2023), belonging to the 2.3.4.4b clade that circulates worldwide [34,35,36,37,38]. The A549 cells expressing different homologous were infected with the indicated virus at a MOI of 0.01. Consistently, overexpressing USP17L13 significantly improved the replication for all tested subtypes, increasing viral titers by 587%, 286%, and 400% for the H1N1, H3N2, and H5N1 viruses, respectively, at 72 h post infection (Figure 2C). These results collectively demonstrate that USP17L13 is a potent and broad-spectrum enhancer of influenza virus replication.

### 3.3. USP17L13 Induces a Proviral State in Host Cells

**Figure 3:**
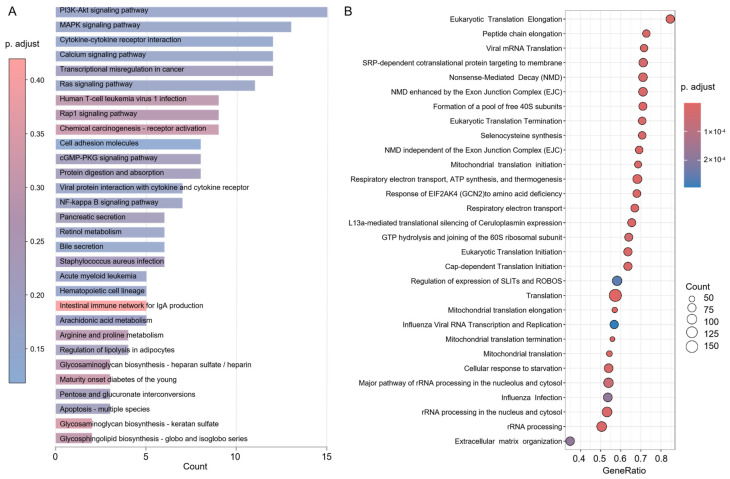
Transcriptional analysis of A549 cells following PR8 virus infection. Cells were infected with PR8 virus at an MOI of 1, and total RNA was collected for RNA-seq analysis. (**A**) KEGG pathway enrichment analysis of downregulated genes in USP17L13-overexpressing A549 cells compared with control cells. The x-axis represents the number of enriched genes, the y-axis indicates the KEGG pathways, and the color indicates the adjusted *p* value. (**B**) Bubble plot showing the GSEA-Reactome results for USP17L13-overexpressing A549 cells compared with control cells. The x-axis represents the GeneRatio, the y-axis indicates the Reactome pathways, bubble size denotes the number of core enriched genes, and bubble color represents the adjusted *p* value.

To figure out the mechanisms by which USP17L13 facilitates influenza virus replication, we performed transcriptomic sequencing on PR8-infected A549 cells with stable USP17L13 overexpression and matched control cells. First, the KEGG pathway enrichment analysis of the downregulated transcripts revealed a profound suppression of host innate immunity, particularly the pathways essential for immune activation (Figure 3A). Notably, key defense pathways, including the phosphoinositide 3-kinase–protein kinase B (PI3K-Akt), mitogen-activated protein kinase (MAPK), and NF-κB pathways, were significantly downregulated, suggesting a systemic failure in cellular defense signaling. Furthermore, pathways involved in cytokine-cytokine receptor interaction and arachidonic acid metabolism were markedly attenuated, indicating that USP17L13 not only suppresses the antiviral response but also disrupts the synthesis of lipid-derived inflammatory mediators. In contrast to the downregulated immune defenses, gene set enrichment analysis (GSEA) of the upregulated genes uncovered a marked upregulation of the host’s biosynthetic and metabolic machinery (Figure 3B). Gene sets associated with the ribosome, including the cytosolic ribosome and cytosolic large ribosomal subunit, as well as oxidative phosphorylation-related mitochondrial processes, were strongly enriched (Figure S2). Crucially, GSEA-Reactome specifically identified “Viral mRNA Translation” and “Influenza Viral RNA Transcription and Replication” as the most active biological processes in USP17L13-overexpressing cells. Together, these findings suggest that USP17L13 weakens host antiviral defense pathways while enhancing cellular biosynthetic and metabolic activities that support efficient viral replication. Accordingly, USP17L13 appears to shift host cells from an antiviral state toward a more virus-permissive state.

### 3.4. USP17L13 Suppresses Antiviral Innate Immune Signaling

**Figure 4:**
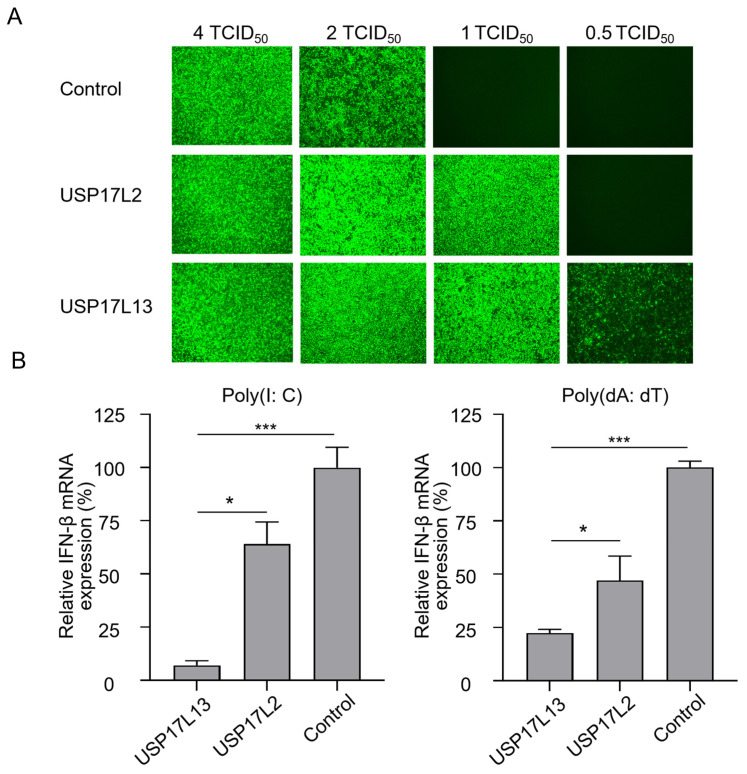
USP17L13 suppresses type I IFN-mediated antiviral responses. (**A**) USP17L13 enhances cellular susceptibility to VSV-GFP infection. A549 cells stably expressing USP17L2, USP17L13, or an empty vector control were infected with the indicated serial dilutions of VSV-GFP. Representative fluorescence microscopy images were captured at 48 h post infection. (**B**) USP17L13 suppresses IFN-β expression. The indicated stable A549 cell lines were stimulated with Poly(I: C) or Poly(dA: dT). Relative mRNA expression levels of IFN-β were analyzed by RT-qPCR and normalized to the vector control group. Data is presented as mean ± SD (*n* = 3). Statistical significance was determined by Independent *t*-test (* *p* < 0.05; *** *p* < 0.001). TCID_50_, 50% tissue culture infectious dose.

A previous study demonstrated that USP17L2 modulates the type I IFN signaling pathway [30], and the RNA-seq data suggest that USP17L13 suppresses the immune response. Therefore, we next investigated whether USP17L13 also has this function. A VSV-GFP reporter system [39], which is particularly sensitive to the antiviral state triggered by IFN, was used to examine IFN production. A549 cells overexpressing USP17L2, USP17L13, or control cells were infected with VSV-GFP at doses of 4, 2, 1, and 0.5 TCID_50_ (50% tissue culture infectious dose). In control cells, viral replication was observed at the higher doses of 4 and 2 TCID_50_. However, overexpression of USP17L2 partially attenuated the antiviral response, allowing successful infection at 1 TCID_50_. Remarkably, USP17L13-expressing cells exhibited the highest susceptibility, supporting viral replication even at a dose as low as 0.5 TCID_50_ (Figure 4A). These data suggest that USP17L13 is a potent negative regulator of the host antiviral response.

To further confirm the role of USP17L13 in immune suppression, we stimulated these cells with Poly(I: C) and Poly(dA: dT), which mimic dsRNA and dsDNA, respectively, and activate the corresponding innate immune signaling pathways. Upon Poly(I: C) treatment, IFN-β mRNA levels in USP17L13- and USP17L2-overexpressing cells were reduced to approximately 7% and 22% of those in control cells, respectively (Figure 4B). Similarly, following Poly(dA: dT) stimulation, IFN-β mRNA levels decreased to approximately 47% and 64% of control levels in USP17L13- and USP17L2-overexpressing cells, respectively (Figure 4B). Collectively, these results demonstrate that USP17L13 is a substantially more potent antagonist of the IFN signaling pathway than USP17L2, thereby impairing immune responses mediated by both RNA and DNA sensing.

### 3.5. USP17L13 Suppresses IFN Signaling by Promoting the Degradation of RIG-I and MDA5

**Figure 5:**
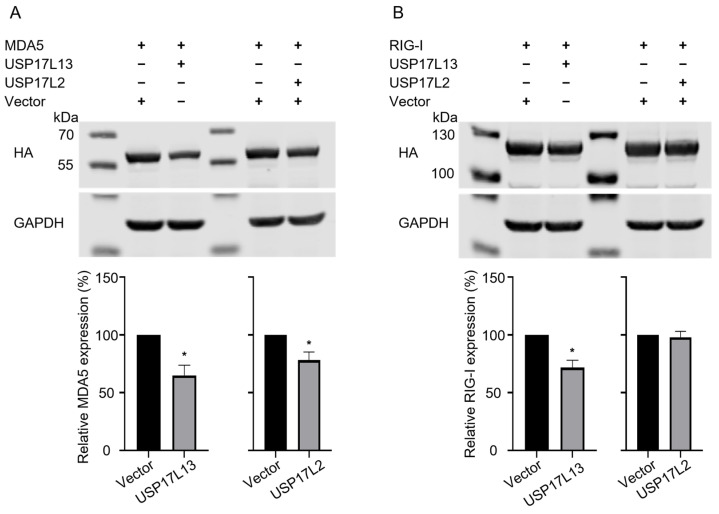
USP17L13 promotes the degradation of RIG-I and MDA5. (**A**,**B**) HEK293T cells were co-transfected with HA-tagged MDA5 (**A**) or RIG-I (**B**) along with either an empty vector, USP17L13, or USP17L2. Cells were harvested 48 h post transfection, and protein levels were analyzed by Western blotting using anti-HA antibodies. Representative images are shown in the upper panels. The lower panels display the densitometric quantification of RIG-I or MDA5 protein levels normalized to GAPDH. Data are presented as the mean ± SD from three independent experiments. Statistical analysis was performed using a two-tailed one-sample Student’s *t*-test (* *p* < 0.05).

To identify the specific factor(s) of the IFN signaling pathway targeted by USP17L13, we co-transfected HEK293T cells with USP17L13 and several key signaling proteins, including RIG-I, MDA5, MAVS, IKKε, and STING. USP17L2, a previously characterized USP17 family member (29), was included as a comparative control. Initial Western blot screening revealed that overexpression of USP17L13 had no observed effect on the protein levels of MAVS, IKKε, or STING compared to the vector control (Figure S3). However, USP17L13 overexpression led to an obvious reduction in the protein levels of both RIG-I and MDA5, suggesting a specific degradative phenotype (Figure S3). For USP17L2, the degradation effect is similar to USP17L13, but no degradation effect on RIG-I (Figure S3). Because USP17L13 exhibited stronger proviral activity than USP17L2 in promoting influenza virus replication (Figure 2B), we next compared their relative abilities to induce the degradation of RIG-I and MDA5. As presented in Figure 5A, USP17L13 induced more pronounced degradation of MDA5 than USP17L2. Notably, USP17L13 also promoted robust degradation of RIG-I, whereas USP17L2 showed little to no effect (Figure 5B). These findings indicate that USP17L13 suppresses IFN signaling by selectively promoting the degradation of RIG-I and MDA5, with a stronger effect on RIG-I than that observed for USP17L2.

### 3.6. USP17L13 Expression Is Induced by IFN and Inflammatory Factors

**Figure 6:**
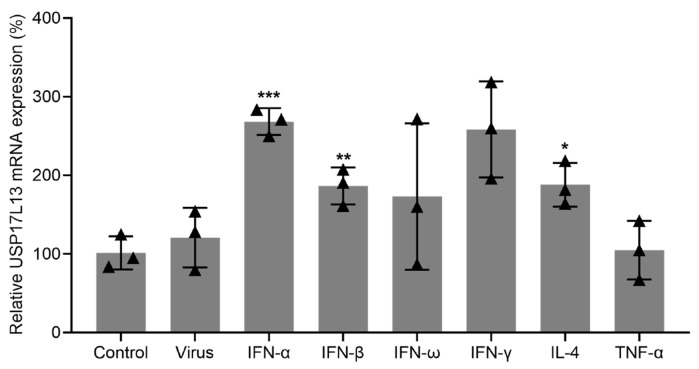
USP17L13 expression is induced by various interferons and cytokines. Relative mRNA levels of USP17L13 were measured by RT-qPCR in A549 cells following treatment with the indicated proteins, namely, type I interferons IFN-α, IFN-β, IFN-ω, type II interferon IFN-γ, and representative inflammatory or immunomodulatory cytokines TNF-α and IL-4, or virus infection. Data are normalized to the untreated control and presented as the mean ± SD (*n* = 3). Statistical significance was determined compared to the control group using Independent *t*-test (* *p* < 0.05; ** *p* < 0.01; *** *p* < 0.001).

To further characterize the transcriptional regulation of USP17L13, we examined its expression following treatment with a panel of immune factors. Because members of the USP17 family are often described as cytokine-responsive genes, we included type I interferons (IFN-α, IFN-β, and IFN-ω), the type II interferon (IFN-γ), and representative inflammatory or immunomodulatory cytokines [tumor necrosis factor-alpha (TNF-α) and interleukin-4 (IL-4)]. As shown in Figure 6, USP17L13 expression was most strongly induced by IFN-α. Significant upregulation was also observed following IFN-γ, IFN-β, and IL-4 treatment. Although IFN-ω increased USP17L13 expression, this effect was not statistically significant. In contrast, TNF-α and virus infection did not significantly alter USP17L13 expression relative to the untreated control. These results suggest that USP17L13 is preferentially induced by interferons and selected inflammatory cytokines.

## 4. Discussion

In this study, we demonstrated that the human deubiquitinase USP17L13 promotes the replication of both human H1N1 and H3N2, as well as avian H5N1 influenza viruses in human A549 cells. Although deubiquitinases are generally considered to stabilize their substrates by removing ubiquitin chains, USP17L13 overexpression instead led to the degradation of RIG-I and MDA5, thereby suppressing IFN production. Interestingly, influenza virus infection did not induce USP17L13 expression, whereas IFN-α, IFN-β, IFN-γ, and IL-4 markedly increased its expression. These findings suggest that USP17L13 may serve as a negative-feedback regulator of innate immune signaling, limiting excessive IFN responses after cytokine stimulation.

According to a previous study [30], USP17L2 overexpression non-specifically removed ubiquitin chains from multiple proteins, including MDA5, RIG-I, MAVS, IKKε, and STING. However, only the protein level of MDA5 was decreased in that study, which is consistent with our data. In our study, we demonstrated that USP17L13 exerts a proviral effect by promoting the degradation of both RIG-I and MDA5, whereas USP17L2 promotes the degradation of MDA5 only (Figure 5). These observations suggest that individual USP17 family members may exert distinct functions despite their high sequence similarity. More specifically, different USP17 homologs may differ not only in substrate preference, but also in how deubiquitination affects substrate stability. One possibility is that USP17L2 and USP17L13 may directly or indirectly remove different types of ubiquitin chains from their substrates. In many studies, K48-linked ubiquitination has been reported to be associated with proteasomal degradation [40,41], whereas K63-linked ubiquitination mainly regulates non-degradative functions, such as protein activation and signal transduction [42]. Another possibility is that USP17L13 may enhance the stability of an unidentified E3 ligase whose substrates are MDA5 and/or RIG-I. For example, an E3 ubiquitin–protein ligase, ring finger protein 125 (RNF125), was found to mediate negative regulation of the immune response via the degradation of RIG-I [43]. Besides this early finding, others also found that E3 ligases such as RNF122 [44], tripartite motif containing 40 (TRIM40) [45,46], and TRIM48 [47] could degrade RIG-I and/or MDA5. These findings suggest that USP17L13 and USP17L2 regulate the IFN pathway through different mechanisms, although the precise mechanism by which USP17L13 acts remains speculative and needs to be investigated.

The USP17 family was originally identified as a group of immediate-early genes induced by cytokine stimulation in mice [48,49]. However, this family appears to be considerably expanded in humans, with 26 distinct homologs in humans compared with only 5 in mice. Bioinformatic analyses have shown that multiple human USP17 family members are located mainly on chromosome 4p16.1 and 8p23.1, with the latter region residing within the copy-number-variable β-defensin cluster [48,49]. These observations suggest that the expansion of the USP17 family may have been associated with functional diversification, allowing different homologs to regulate distinct biological processes. Indeed, USP17L2 has been reported to regulate innate immune responses and to be involved in multiple cancers [27,28,29,49], whereas USP17L13 remains largely uncharacterized. Notably, our bioinformatic analyses suggested that USP17L13 is expressed at very low levels or is undetectable in publicly available datasets from healthy and diseased human samples. Considering that USP17L13 was not upregulated by influenza virus infection in vitro but was markedly upregulated by IFN-α, IFN-β, IFN-γ, and IL-4, it is likely that USP17L13 functions primarily as a cytokine-responsive negative regulator of innate immunity acting to restrain excessive IFN signaling after cytokine stimulation induced by virus infection.

This study has several limitations. First, although our results show that USP17L13 inhibits IFN production by promoting the degradation of RIG-I and MDA5, influenza virus infection is sensed not only by cytoplasmic RLRs but also by endosomal RNA sensors, including Toll-like receptor 3 (TLR3) and TLR7 [50]. Previous work on USP17L2 reported that its knockdown had little effect on TLR3-mediated signaling [30], raising the possibility that USP17 family members may mainly act on RLR-dependent pathways. However, whether this is also the same for USP17L13 remains unclear. It will therefore be important to determine whether USP17L13 has any effect on TLR3- or TLR7-mediated responses during influenza virus infection. Second, USP17L13 showed very low basal expression in our analyses, and the biological effect of its complete loss remains unknown. In cells, influenza virus infection alone did not markedly induce USP17L13 expression, even though viral infection is expected to trigger cytokine production. One possible explanation is that virus infection in A549 cells may not fully reproduce the immune environment that occurs in vivo. In infected tissues, cytokines and chemokines are produced by multiple cell types, and their combined effects may differ from those observed in a simplified cell culture system. This may be particularly important during co-infection or superinfection with different respiratory viruses, where RLR, TLR, and cytokine signaling pathways may be activated simultaneously or sequentially. Under such conditions, USP17L13 may be more strongly induced and may influence the balance between antiviral defense, viral replication, and cytokine-mediated inflammation. Future loss-of-function studies, especially those using USP17L13 knockout animals, will be needed to clarify its biological role in vivo.

Beyond its role in innate immune regulation, USP17L13 may also have potential practical value. Because USP17L13 enhanced the replication of multiple influenza viruses, it may provide a basis for developing high-yield cell lines for virus production. Such an approach could be useful for improving the efficiency and scalability of influenza vaccine manufacturing. However, this possibility still needs to be tested in established production platforms.

## 5. Conclusions

This study identifies USP17L13 as a new negative regulator of the innate immune response. These findings provide a rationale for exploring host-directed therapeutic strategies to alleviate excessive inflammation and immune-mediated pathology during influenza virus infection, and may also have potential biotechnological applications.

## Data Availability

All data are provided as main figures or Supplementary Materials within the paper.

## Supplementary Materials

The following supporting information can be downloaded at: https://www.mdpi.com/article/10.3390/v18050575/s1, Figure S1: Gene ontology (GO) enrichment analysis of differentially expressed genes; Figure S2: Bubble plot showing the top GO terms enriched in USP17L13-overexpressing cell; Figure S3: Degradative effects of USP17L13 on key factors in the IFN signaling pathway; Table S1: Up-regulated gene in this study; Table S2: Up-regulated genes in King et al.’s study; Table S3: Differentially expressed genes between PR8-infected USP17L13-overexpressing and control A549 cells.

## Abbreviations

The following abbreviations are used in this manuscript:

RIG-I	Retinoic acid-inducible gene I
RLR	The retinoic acid-inducible gene I (RIG-I)-like receptor
USP17L13	Ubiquitin carboxyl-terminal hydrolase 17-like protein 13
IFN	interferon
NF-κB	nuclear factor kappa B
MDA5	melanoma differentiation-associated protein 5
PRRs	pattern recognition receptors
DUBs	deubiquitinating enzymes
MAVS	mitochondrial antiviral-signaling protein
TRAF3	tumor necrosis factor receptor-associated factor 3
NP	Nucleoprotein
PB2	Polymerase basic protein 2
GO	gene ontology
BP	biological process
MF	molecular function
CC	cellular component
MOI	multiplicity of infection
GSEA	gene set enrichment analysis
TCID_50_	50% tissue culture infectious dose
MAVS	Mitochondrial Antiviral Signaling Protein
IKKε	inhibitor of nuclear factor κ-B kinase subunit ε
STING	stimulator of interferon genes
MDCK	Madin–Darby canine kidney
DMEM	Dulbecco’s modified Eagle’s medium
NCS	newborn calf serum
FBS	fetal bovine serum
VSV	Vesicular stomatitis virus
TPCK	L-1-tosylamido-2-phenylethyl chloromethyl ketone-treated trypsin
PBS	Phosphate-buffered saline
GAPDH	Glyceraldehyde-3-phosphate dehydrogenase
