# Supplementary material for: USP17L13 Enhances Influenza a Virus Replication by Mediating the Degradation of RIG-I and MDA5
Source: Viruses. 2026 May 20;18(5):575. doi: 10.3390/v18050575 (PMC13211412; doi:10.3390/v18050575)
Supplement: Supplementary file 1 [file viruses-18-00575-s001.zip › Supplementary Files/Figures S1–S3 20260413.pptx]

## Slide 1
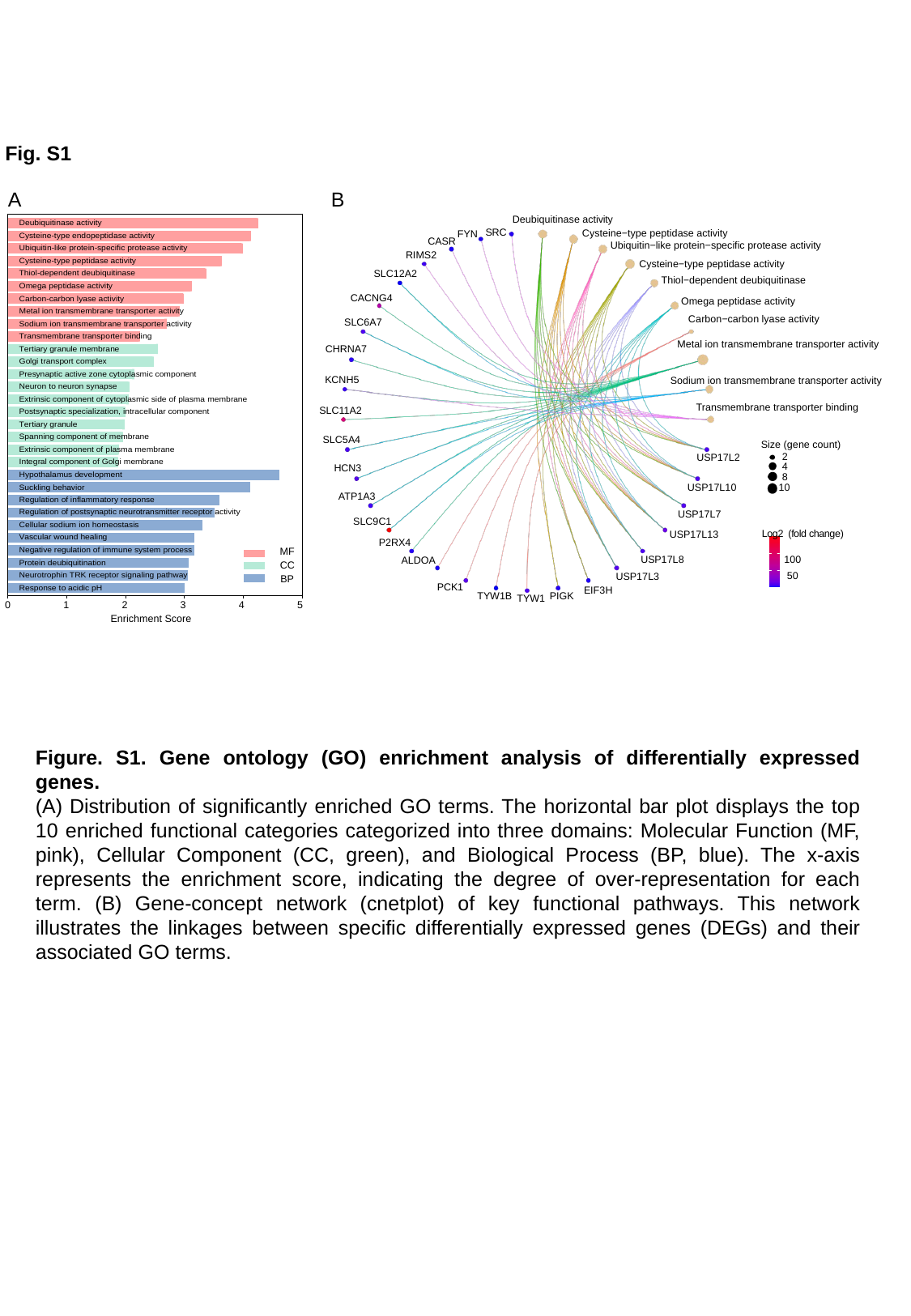

Fig. S1
A
B
0
1
2
3
4
5
Enrichment Score
 Deubiquitinase activity
SRC
Cysteine−type peptidase activity
FYN
CASR
Ubiquitin−like protein−specific protease activity
RIMS2
Cysteine−type peptidase activity
SLC12A2
Thiol−dependent deubiquitinase
CACNG4
Omega peptidase activity
SLC6A7
Carbon−carbon lyase activity
Metal ion transmembrane transporter activity
CHRNA7
KCNH5
 Sodium ion transmembrane transporter activity
SLC11A2
 Transmembrane transporter binding
SLC5A4
Size (gene count)
2
4
8
10
USP17L2
HCN3
USP17L10
ATP1A3
Log2 (fold change)
100
50
USP17L7
SLC9C1
USP17L13
P2RX4
USP17L8
ALDOA
USP17L3
PCK1
EIF3H
TYW1B
PIGK
TYW1
MF
CC
BP
Figure. S1. Gene ontology (GO) enrichment analysis of differentially expressed genes.
(A) Distribution of significantly enriched GO terms. The horizontal bar plot displays the top 10 enriched functional categories categorized into three domains: Molecular Function (MF, pink), Cellular Component (CC, green), and Biological Process (BP, blue). The x-axis represents the enrichment score, indicating the degree of over-representation for each term. (B) Gene-concept network (cnetplot) of key functional pathways. This network illustrates the linkages between specific differentially expressed genes (DEGs) and their associated GO terms.

## Slide 2
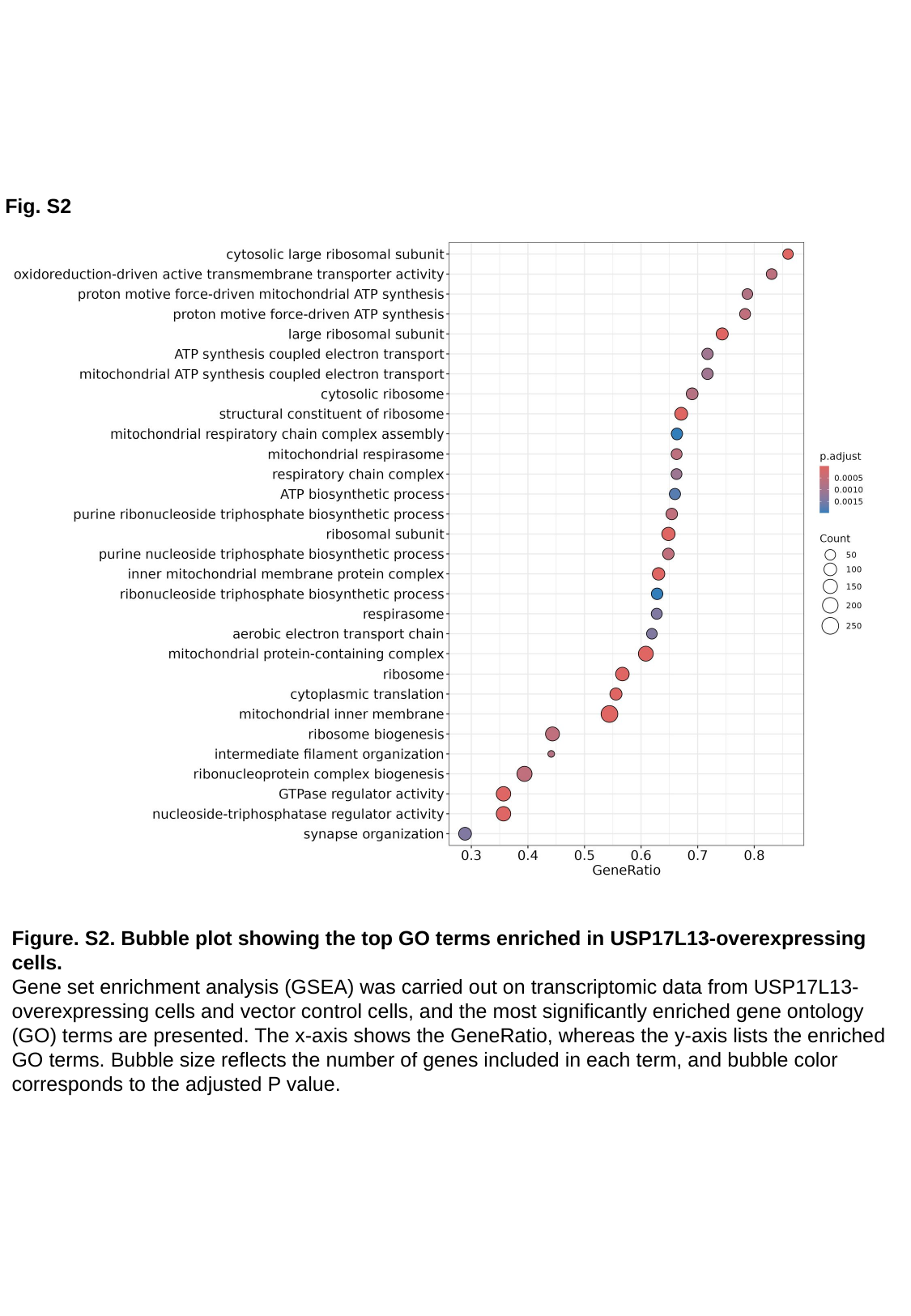

Fig. S2
Figure. S2. Bubble plot showing the top GO terms enriched in USP17L13-overexpressing cells.
Gene set enrichment analysis (GSEA) was carried out on transcriptomic data from USP17L13-overexpressing cells and vector control cells, and the most significantly enriched gene ontology (GO) terms are presented. The x-axis shows the GeneRatio, whereas the y-axis lists the enriched GO terms. Bubble size reflects the number of genes included in each term, and bubble color corresponds to the adjusted P value.

## Slide 3
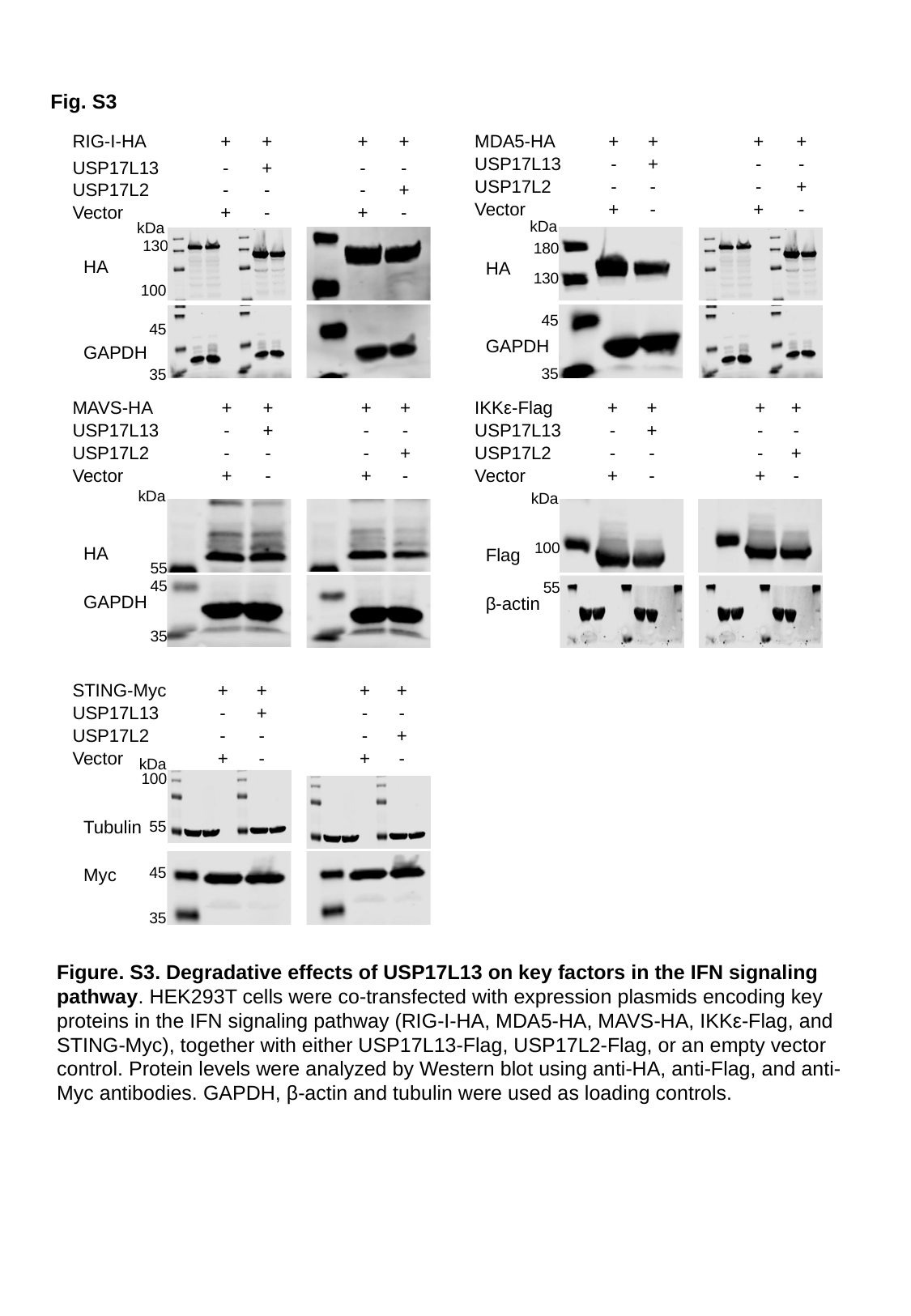

Fig. S3
| RIG-I-HA | + | + | | + | + |
| --- | --- | --- | --- | --- | --- |
| USP17L13 | - | + | | - | - |
| USP17L2 | - | - | | - | + |
| Vector | + | - | | + | - |
| MDA5-HA | + | + | | + | + |
| --- | --- | --- | --- | --- | --- |
| USP17L13 | - | + | | - | - |
| USP17L2 | - | - | | - | + |
| Vector | + | - | | + | - |
kDa
kDa
130
100
45
35
180
130
45
35
HA
HA
GAPDH
GAPDH
| MAVS-HA | + | + | | + | + |
| --- | --- | --- | --- | --- | --- |
| USP17L13 | - | + | | - | - |
| USP17L2 | - | - | | - | + |
| Vector | + | - | | + | - |
| IKKε-Flag | + | + | | + | + |
| --- | --- | --- | --- | --- | --- |
| USP17L13 | - | + | | - | - |
| USP17L2 | - | - | | - | + |
| Vector | + | - | | + | - |
kDa
kDa
100
55
HA
Flag
55
45
35
GAPDH
β-actin
| STING-Myc | + | + | | + | + |
| --- | --- | --- | --- | --- | --- |
| USP17L13 | - | + | | - | - |
| USP17L2 | - | - | | - | + |
| Vector | + | - | | + | - |
kDa
100
55
45
35
Tubulin
Myc
Figure. S3. Degradative effects of USP17L13 on key factors in the IFN signaling pathway. HEK293T cells were co-transfected with expression plasmids encoding key proteins in the IFN signaling pathway (RIG-I-HA, MDA5-HA, MAVS-HA, IKKε-Flag, and STING-Myc), together with either USP17L13-Flag, USP17L2-Flag, or an empty vector control. Protein levels were analyzed by Western blot using anti-HA, anti-Flag, and anti-Myc antibodies. GAPDH, β-actin and tubulin were used as loading controls.
